# Correction: Internalizing symptoms and family functioning predict adolescent depressive symptoms during COVID-19: A longitudinal study in a community sample

**DOI:** 10.1371/journal.pone.0280899

**Published:** 2023-01-20

**Authors:** The PLOS ONE Staff

## Notice of republication

This article was republished on December 29, 2022, to correct for errors in the Data Availability statement. The publisher apologizes for the errors. Please download this article again to view the correct version. The originally published, uncorrected article and the republished, corrected article are provided here for reference.

## Supporting information

S1 File Originally published, uncorrected article. (PDF)

S2 File Republished, corrected article. (PDF)
