# Supplementary material for: Internalizing symptoms and family functioning predict adolescent depressive symptoms during COVID-19: A longitudinal study in a community sample
Source: PLoS One. 2022 Mar 18;17(3):e0264962. doi: 10.1371/journal.pone.0264962 (PMC8932580; doi:10.1371/journal.pone.0264962)
Supplement: Attachment — Submitted filename: Response to Reviewers.docx [file pone.0264962.s001.docx]

**Editor Comments:**

1. In the Methods it is stated that data were collected in two waves: please explain when exactly.

**We clarified this on p.6 lines 265-266.**

2. In the Results the following is stated:

In 2019, internalizing and externalizing symptoms were 10 and 16% above the clinical cut-off,
respectively, whereas during the lockdown, depressive symptoms were 24% above the cut-off
(Table 1). Please add the % of internalizing and externalizing symptoms above the clinical cut-off in 2020, as well as the % that showed depressive symptoms above clinical cut-off in 2019 – to allow more precise comparisons.

**In this study, we assessed internalizing/externalizing symptoms prior to the pandemic and depression symptoms during the pandemic. Therefore, the percentages of internalizing/externalizing during the pandemic, and the percentage of depressive symptoms prior to the pandemic are not available.**

3. In the Discussion is stated:

Although we only assessed internalizing symptoms, and not depressive symptoms, prior
to the lockdown….

Why didn’t you study the specific relationship between prior depressive symptoms and depressive symptoms during the lockdown? Please explain either in the Discussion or clarify your research questions in this regard in the Introduction.

**This study stems from a longitudinal study that started in late pregnancy and followed children across development (BIBO study). The aim of the BIBO study was to examine early predictors of child behavioral development. For this reason, and also because depressive symptoms are less common in early/middle childhood, we examined internalizing and externalizing behavior problems as more broader, overarching constructs of behavior. However, amidst the COVID-19 outbreak, we were worried about deterioration of mental health, especially also as our children had reached early adolescence, an age in which more children start to develop depressive symptoms. That our assessments of behavior were not identical at both waves is now included as a limitation to the Discussion section (pp. 14-15, lines 480-489).**

4. You also state:

It could be that adolescents from high educated, wealthy and low risk families may be even less prepared to face a challenging crisis than adolescents who have previously experienced hardship. How does this idea relate to your sample? Please explain.

**We added an explanation of this idea to the Discussion (pp. 11-12, lines 396-403). We additionally took out the word ‘wealthy’ to characterize the families as this was an assumption not based on actual financial data.**

**Reviewer #1:**

1. I think the first paragraph is too long. Could it be split in two more homogeneous paragraphs? Or maybe just cut shorter?

**We have shortened the first paragraph accordingly (p. 3).**

2. Line 63: I would say that life events are not “entered”, but experienced.

**We changed the wording as suggested (p. 3, line 59).**

3. Lines 75-77: the aim can be written a bit more clearly: will you be looking at changes in symptoms pre- to during the first lockdown, or to moderation by pre-existing symptoms? The two previous sentences (lines 72-74) imply testing mean level changes, as they offer contrasting extant evidence for increase or decrease in symptoms. Please clarify the aim.

**We have now clarified the aim (p. 4, lines 82-83)**.

4. Lines 78-94: I think this paragraph is a bit confusing, as ideas regarding parenting and parent-adolescent relationship quality are intertwined and likely used interchangeably with family systems ideas. I think these two (parenting/parent-adolescent relationship quality and family functioning) are quite distinct concepts. Indeed, in my view family systems theories actually argue for this exact topic: how the system functions as a whole is not the same as how individuals behave (parenting) or how sub-systems within the system (e.g., the parent-adolescent dyad) interact. Please clarify.

**We thank the reviewer for pointing this out and have rewritten this paragraph to highlight the distinction between parenting/parent-adolescent relationships aspects, and family functioning (p. 4, lines 87-98).**

5. Related to the above: If your family functioning measure has separate dimensions taping onto family as a system *and* parenting or p-a relationships more specifically, then it would be interesting to see what works best as a buffer: is it the system as a whole, or the parenting more specifically that might buffer during crises like the COVID-19?

**This is a very interesting question. Given the rationale of the study to look at family functioning in general, and not specific sub-components, in combination with the lack of sufficient power, we did not run additional analyses with the subdomains of family-related aspects. We added these research questions as venues for future research to the discussion section, in combination with the outcomes of our exploratory analyses. The results of these analyses revealed significant main effects of roles, affective responsiveness and affective involvement (p. 13, lines 441-448).**

6. Lines 90-94: please consider reporting contrasting or clarifying evidence for the potential effects (or absence thereof) of family functioning on adolescent mental health, e.g. Mastrotheodoros et al., 2020 [https://doi.org/10.1007/s10964-019-01094-z](about:blank)

**We thank you for pointing us to this work, which we have now added to our introduction (p. 4, lines 95-96).**

7. Lines 116-118: why would you hypothesize that adolescents with higher pre-existing externalizing would show higher internalizing? This comes a bit as a surprise, given the preceding introduction. A bit more clarity here would be welcome. Consider taking into account literature on the general psychopathology (p) factor for the possible interrelations between internalizing and externalizing.

**We now clarify in our hypothesis that we expect that either internalizing or externalizing symptoms may be linked to higher depressive symptoms during the pandemic, in line with previous reports showing a positive relation between internalizing/externalizing symptoms and depression symptoms (e.g. Vinnakota & Kaur, 2018) (pp. 65-, lines 249-259).**

8. Line 127: I get confused by the mean age reported here, compared to the one reported on line 124. Please clarify.

**We clarified the mean age and sd for the children at each assessment point (p. 6, lines 265-266).**

9. Lines 163-164: it looks like the citation (nr. 36) is wrong? I understand that this is a scale developed recently, but the reference is from 1997?

**We corrected this accordingly on p. 8, line 305.**

10. Lines 190-191: maybe good to remind the reader that those percentages come from different scales?

**We highlighted now that the percentages reported stem from two different scales (p. 9, lines 331-334).**

11. Lines 197-198: girls have higher scores, but the r correlations are negative. This is slightly confusing, not only because of the sign (a “higher” is indicated by a negative sign), but also because of the test statistic. Why r and not t-test? Maybe I am missing something here.

**We clarified the statistical test used to quantify the relation between the sex variable and internalizing or depressive symptoms and its interpretation in the descriptive analyses section. The sign depends on how sex was coded (0 or 1) and r refers to the correlation coefficient between the sex variable and internalizing or depressive symptoms. For clarity, we also added the t-test values (p. 9, lines 339-342).**

12. Lines 246-248: maybe good to remind the reader that different measures were applied pre- and during the pandemic.

**We have added that these symptoms were assessed with different instruments (p.11, lines 391-393).**

13. Line 267: “across the lifespan” is a bit far-stretched, as there were only data from ages 13-14 in this study.

**We deleted this phrasing, p. 12, line 419 and p. 13, line 425.**

14. Lines 277-285: I think the evidence from the study suggested above (Mastrotheodoros et al., 2020) is also important here. In that study we found no evidence that family functioning has within-person effects on adolescent psychological symptoms, neither was there evidence for child mental health effects on family functioning. It might be that family functioning and adolescent mental health are only related on the between-person level. Given the results of that study, it would be better to avoid using causal language (line 278: “alleviate”), and keep the interpretation of your results on the between-person level.

**We thank you for pointing this out to us. We’ve now rephrased causal language where indicated and throughout the manuscript.**
